# Diatom aggregation when exposed to crude oil and chemical dispersant: Potential impacts of ocean acidification

**DOI:** 10.1371/journal.pone.0235473

**Published:** 2020-07-07

**Authors:** Jennifer L. Genzer, Manoj Kamalanathan, Laura Bretherton, Jessica Hillhouse, Chen Xu, Peter H. Santschi, Antonietta Quigg

**Affiliations:** 1 Department of Marine Biology, Texas A&M University at Galveston, Galveston, Texas, United States of America; 2 Department of Oceanography, Dalhousie University, Halifax, Nova Scotia, Canada; 3 Department of Marine Science, Texas A&M University at Galveston, Galveston, Texas, United States of America; Luleå University of Technology, SWEDEN

## Abstract

Diatoms play a key role in the marine carbon cycle with their high primary productivity and release of exudates such as extracellular polymeric substances (EPS) and transparent exopolymeric particles (TEP). These exudates contribute to aggregates (marine snow) that rapidly transport organic material to the seafloor, potentially capturing contaminants like petroleum components. Ocean acidification (OA) impacts marine organisms, especially those that utilize inorganic carbon for photosynthesis and EPS production. Here we investigated the response of the diatom *Thalassiosira pseudonana* grown to present day and future ocean conditions in the presence of a water accommodated fraction (WAF and OAWAF) of oil and a diluted chemically enhanced WAF (DCEWAF and OADCEWAF). *T*. *pseudonana* responded to WAF/DCEWAF but not OA and no multiplicative effect of the two factors (i.e., OA and oil/dispersant) was observed. *T*. *pseudonana* released more colloidal EPS (< 0.7 μm to > 3 kDa) in the presence of WAF/DCEWAF/OAWAF/OADCEWAF than in the corresponding Controls. Colloidal EPS and particulate EPS in the oil/dispersant treatments have higher protein-to-carbohydrate ratios than those in the control treatments, and thus are likely stickier and have a greater potential to form aggregates of marine oil snow. More TEP was produced in response to WAF than in Controls; OA did not influence its production. Polyaromatic hydrocarbon (PAH) concentrations and distributions were significantly impacted by the presence of dispersants but not OA. PAHs especially Phenanthrenes, Anthracenes, Chrysenes, Fluorenes, Fluoranthenes, Pyrenes, Dibenzothiophenes and 1-Methylphenanthrene show major variations in the aggregate and surrounding seawater fraction of oil and oil plus dispersant treatments. Studies like this add to the current knowledge of the combined effects of aggregation, marine snow formation, and the potential impacts of oil spills under ocean acidification scenarios.

## Introduction

The Deepwater Horizon (DwH) blowout occurred on April 20, 2010, killing 11 people, and leaking crude oil and natural gas over 87 days, resulting in an estimated 4.1 million barrels of Louisiana Sweet Oil entering the Gulf of Mexico [1, 2]. In attempts to mitigate the effects of the oil spill on the surface of the ocean, coastline and marshes, an estimated 37,500 barrels of dispersant (Corexit), more than during any previous spill, was applied at surface and at depth [3]. In the weeks following the DwH oil spill, large, fast sinking marine snow was observed [4, 5, 6, 7]. When oil is present in the environment, marine oil snow (MOS) sedimentation and flocculent accumulation (MOSSFA) is a natural mechanism for transporting the oil to the seafloor [4, 5, 6]. This MOSSFA phenomenon has been intensively studied as it is thought to have significantly contributed to bioremediation of the oil [e.g., 4, 5, 6, 7], with calculations suggesting up to 31% was returned to the seafloor [8, 9, 10, 11]. Previous work has shown that phytoplankton responses towards oil and/or oil plus dispersant are species-specific [e.g., 12, 13]. The relative abundance of diatoms actually increased following DwH [14]. They were found to be significant contributors to the MOS [14, 15, 16], making this an important group to focus on in oil spill-related studies.

Phytoplankton, the primary drivers of the biological carbon pump [e.g., 17], are responding to the downward shift in seawater pH associated with ocean acidification (OA) [18, 19, 20]. Before the Industrial Revolution, the ocean’s pH was 8.2; it is projected to reach 7.9 by the end of this century [18,19]. To date, the pH of the surface ocean has dropped to 8.1, which on a logarithmic scale is a 25% change with long-term effects [18,19]. Concurrently, climate change is altering ocean temperatures, stratification, nutrient availability and cycling, and ultimately phytoplankton primary productivity and community composition [21, 22, 23]. Diatoms, a group of phytoplankton responsible for ~40% of marine inorganic carbon fixation [24, 25], exude extracellular polymeric substances (EPS), some of which form transparent exopolymer particles (TEP) [5, 6, 26, 27, 28, 29]. These exudates mix with particulates and other organic materials to form marine snow, which is ubiquitous throughout the marine environment and responsible for the majority of downward carbon transport [17, 30, 31, 32, 33]. Changes in the composition and amount of exudates released by microbes as a result of variation/stimuli from their surrounding environment, may ultimately affect the carbon flux, as was observed after the DwH oil spill. Indeed, Riebesell et al. [34] speculated that more TEP may be released in future ocean conditions, and could potentially result in increased aggregation. Others have found that because TEP are buoyant particles, a higher proportion accumulated within aggregates slows their sinking rates, so increased TEP may not actually increase the carbon flux [35]. TEP will stay suspended in surface waters unless the particles attach to heavier materials (fecal pellets, mineral ballast, particulates) [36]. The stickiness of these macromolecules may be affected by changing ocean conditions [29, 35], so there is the potential for compounding effects to the biological carbon pump.

Larger diatoms may be limited in future oceans with increased acidification and declining nutrient availability, potentially leading to a shift towards smaller species [22, 37]. *Thalassiosira pseudonana*, a small and globally abundant diatom often used as a model species, does not appear to be impacted by OA in its growth or photosynthesis [25, 38, 39]. This may be due to its use of a carbon concentrating mechanism, or its small size (2–10 μm) and high surface area to volume ratio which facilitates diffusion of CO_2_ into the cell. Yet, other studies have found *T*. *pseudonana* responds to OA by altering its physiological behavior [40, 41]. Further, OA does affect aggregation processes in this and other species [23, 35, 42]; with precursor concentrations being important [26, 28, 43].

Hence, how phytoplankton will respond to oil spills in the context of ocean acidification is of great interest, i.e., if the OA and oil spills have a synergistic, antagonistic or additive effect on the biological carbon pump, and in particular, MOSSFA-facilitated oil transport. This study used a multifactorial laboratory experiment to investigate the diatom *T*. *pseudonana*. Six treatments: (i) Control with current *p*CO_2_ levels (400 ppm), (ii) an elevated *p*CO_2_ representative of future conditions (750 ppm), (iii) a water accommodated fraction (WAF) of oil, (iv) WAF prepared with elevated *p*CO_2_ seawater (OAWAF), (v) diluted chemically enhanced WAF (DCEWAF), and (vi) DCEWAF prepared with elevated *p*CO_2_ (OADCEWAF). Roller table experiments were performed with cultures of *T*. *pseudonana* grown in exponential growth phase, and EPS, TEP and marine snow formation were measured along with the oil composition.

## Methods

All glassware, roller tanks and culture bottles, were acid washed. Equipment exposed to oil was cleaned sequentially with dichloromethane (DCM), methanol and DI water to remove residual oil compounds.

### Culturing

*Thalassiosira*. *pseudonana* (CCMP 1335; National Center for Marine Algae) was grown in natural seawater collected from the Gulf of Mexico and enriched with f/2 + Si nutrients [44, 45] after autoclaving (121°C, 30 min). One month prior to the experiments, *T*. *pseudonana* was transferred into artificial seawater media [46] prepared with a few modifications. The deionized (DI) water was not sterilized in the autoclave in order to avoid altering the carbonate system [23]. The pH was adjusted to 8.1 using Trizma base. Cultures were maintained at an irradiance of 100–130 μmol m^-2^ s^-1^ and kept at 19 ± 1°C under a 12h:12h light:dark cycle and grown in exponential growth for each of the experiments.

### Carbonate chemistry

Three days prior to starting a roller table experiment, cultures were split into Controls and elevated *p*CO_2_ treatments [38]. Based on previous studies [38, 39], it has been shown that 3 days is sufficient for exponentially growing *T*. *pseudonana* to acclimate changes in *p*CO_2_ concentrations. The pH was maintained at 8.1 for Control and 7.9 for elevated *p*CO_2_ cultures according to Gattuso and Lavigne [47]. The carbonate chemistry was measured according to Seebah et al. [23] after pH and total alkalinity (TA) were determined using a calibrated Accumet AB15 pH meter. The Seacarb R package (version 3.1.1) and CO2SYS [48] were used to calculate *p*CO_2_ levels using salinity, temperature, pH, and TA (https://CRAN.R-project.org/package=seacarb) (Table 1).

**Table 1:** Initial (start of the roller table experiments) carbonate chemistry. Outputs from both CO2SYS and Seacarb R for pCO_2_ (ppm), TCO_2_ (μmol kg SW^-1^), HCO^3-^ (μmol kg SW^-1^), CO_3_^2-^ (μmol kg SW^-1^), and CO2 (μmol kg SW^-1^) are provided.

	Measurement	Control	OA	WAF	OAWAF	DCEWAF	OADCEWAF
Target	pH	8.1	7.9	8.1	7.9	8.1	7.9
	*p*CO_2_	400	750	400	750	400	750
Measured	pH	8.1	7.9	8.1	7.9	8.17	7.99
	TA	2450	2450	2420	2420	2485	2485
CO2SYS	*p*CO_2_	381	651	376	643	318	521
	TCO_2_	2175	2275	2148	2246	2167	2266
	HCO_3_	1965	2118	1940	2092	1928	2086
	CO_3_	197	134	195	132	227	162
	CO_2_	12	22	1	21	10	17
Seacarb R	*p*CO_2_	381	651	376	643	318	521

### Roller tanks

The morning prior to starting experiments, cell counts were performed to determine the volume of *T*. *pseudonana* to add to the roller tanks so the final cell density would be ~10,000 cells mL^-1^. The cylindrical glass roller tanks were carefully filled with cell cultures and treatment media as to avoid air bubbles. Triplicate tanks were prepared for each of the six treatments (18 roller tanks total). Immediately before closing the tanks, bicarbonate and acid were added to reach desired elevated *p*CO_2_ conditions [23, 47]. Tanks were processed individually after three days of continuously rolling (3 turns/min; 19 ± 3°C, in darkness) to simulate sinking through the water column [49]. From each roller tank, the aggregate slurry (AGG) and the surrounding seawater (SSW) were collected and either immediately processed or stored at -20°C.

### Oil and dispersant treatments

The WAF was prepared in a continuous flow-through baffled recirculating tank system [50] filled with artificial seawater (120 L) and Macondo surrogate oil (0.4 mL L^-1^). This was mixed for 24 hrs in the dark before being transferred into the roller tanks. Corexit was pre-mixed with the oil (Corexit-to-oil ratio of 1:20) in order to prepare the DCEWAF following similar procedures to WAF except small (9 L) aspirator bottles were used as smaller volumes were required. The concentrations of oil in the WAF and DCEWAF at the start of the experiments, as estimated oil equivalents (EOE), were measured using a RF-5301 PC Spectrofluorophotometer (322 nm excitation, 376 nm emission) according to Wade et al. [51], with modifications as described in [12, 13]. EOE concentration was determined using a five-point calibration curve prepared with the Macondo surrogate oil (0–5 mg L^-1^).

Polycyclic aromatic hydrocarbons (PAHs) analysis was performed on AGG and SSW samples collected at the end of the experiment. Prior to PAH extraction, each sample was spiked with 50 μL of internal standards (d8-naphthalene, d10-acenaphthene, d10-phenanthrene, d12-chrysene and d12-perylene). SSW samples (700 mL) and AGG samples (5 mL) were extracted with DCM and analyzed with GC/MS as described previously [52, 53]. The concentrations of oil used in this study were well below the 200 mg L^-1^ limit to simulate authentic spill conditions, as this has been a critique among some recent oil toxicity work [50]. Further, the DCEWAF treatments were prepared with EOEs more dilute than those used in previous studies [12, 13, 50] for two reasons. First, efforts were made to manipulate a similar oil concentration in WAF and DCEWAF, the latter of which, if not diluted, could be one to two orders of magnitude higher than WAF. Second, it has been documented that *T*. *pseudonana* is very sensitive to high concentrations of oil in DCEWAF [12]. These treatments were designed to be effective at initiating a response from the diatom but at a dose low enough to be environmentally relevant.

### Biomass

Chlorophyll (chl) *a* was measured as a proxy for diatom biomass according to EPA Method 445 [54]. Both SSW and AGG samples were filtered onto 0.7 μm GF/F and stored at -20°C. Pigment was extracted overnight in the dark at 4°C with a 40:60 dimethyl sulfoxide: 90% acetone solution and then measured using a benchtop 10AU Turner Designs fluorometer. A chlorophyll standard extracted from the alga *Anacystis nidulans* (Sigma—Aldrich) was used to prepare a standard curve.

### Aggregate analysis

SSW and AGG fractions were separated, harvested, and processed as described in detail in Genzer [55]. Tanks were removed from the rollers and the total time for aggregates to settle recorded. Tanks were placed on gridline paper and photographs taken for size analysis using ImageJ software (Version 1.51j8). Photos were converted to greyscale and individual aggregate size was calibrated against the gridline paper. Each of the individual aggregates was outlined using the Polygon Selections tool (400-x magnification), and assessed for a detailed aggregate morphology using area, grey scale color density, perimeter, length and width. These qualitative descriptions were converted into quantitative measures by determining the mean grey value as an optical density unit (ODU) for each aggregate [55]. ImageJ aggregate area measurements from experiment were processed using RStudio (Version 0.99.903) to create an agglomerative dendrogram with limited bias for size classes. Four size classes were identified using this technique: Large (>4.683 cm^2^), Medium (1.551 to 4.682 cm^2^), Small (0.591 to 1.550 cm^2^), and Extra Small (XS) (<0.590 cm^2^) [55]. These size classes were then applied to the analysis of the aggregates. Equivalent spherical diameter (ESD) measurements [56] are commonly used in marine snow studies to normalize aggregate size and shape [4] and determine sinking velocity [15].

### Extracellular materials

TEP analysis was only performed on Control and WAF samples according to Passow and Alldredge [57] as Corexit interferes with the assay [58]. SSW samples and blanks were filtered, stained with Alcian Blue, and stored at -20°C for shipment to the University of California at Santa Barbara (UCSB) for processing. Filters were extracted in 80% H_2_SO_4_ for 2 hrs, and the extract was measured at 787 nm on a Genesys 10S UV-Vis spectrophotometer. A Gum Xanthan (Gxan) equivalents curve was prepared to determine TEP concentrations.

EPS was measured as the sum of proteins and carbohydrates in the AGG (0.5 mL) and SSW (200 mL) at the end of the roller table experiments. These correspond to different pools of EPS, herein called particulate and colloidal, respectively. Samples were filtered using ultra-centrifugal membranes (3 kDa) to collect the particulate fraction of the AGG and the colloidal fraction of SSW, triple rinsed with 18 MΩ –cm Milli-Q Type I water, and concentrated to 2 mL. Proteins were measured using a modified bicinchoninic acid method [59] and a bovine serum albumin (ThermoFisher, Pierce protein assay kit) standard. Neutral sugar concentrations were measured with a glucose standard following the anthrone method [60], and uronic acids were measured with a glucuronic acid (Sigma, CAS 6556-12-3) standard [61]. The sum of neutral sugars and uronic acids was used to calculate carbohydrate concentrations [62]. The ratio of proteins to carbohydrates (P/C) in organic carbon equivalents (i.e., a 40% of neutral sugar mass as organic carbon, 37% of uronic acid, and 53% of protein content) [62] was used to assess the stickiness (coagulation efficiency) of the material [29].

### Statistical analysis

Data is presented as mean ± standard deviation. All statistical tests were performed using built-in RStudio (Version 0.99.903) functions with spreadsheets built in Excel 2016. Student’s *t*-tests were used to compare measurements between two treatments with 95% confidence. Two-way Analysis of Variance Model (ANOVA), which utilized the Tukey’s ‘Honest Significant Difference’ method, compared a measurement between all treatments. *P*-values <0.05 were considered significant for both tests. PAH composition across all the treatments in different fractions were visualized via non-metric dimensional scaling (NMDS) and the similarities in compositions were analyzed with ANOSIM and SIMPER available through vegan package using RStudio.

## Results

### *T*. *pseudonana* aggregation behavior

**Fig 1:**
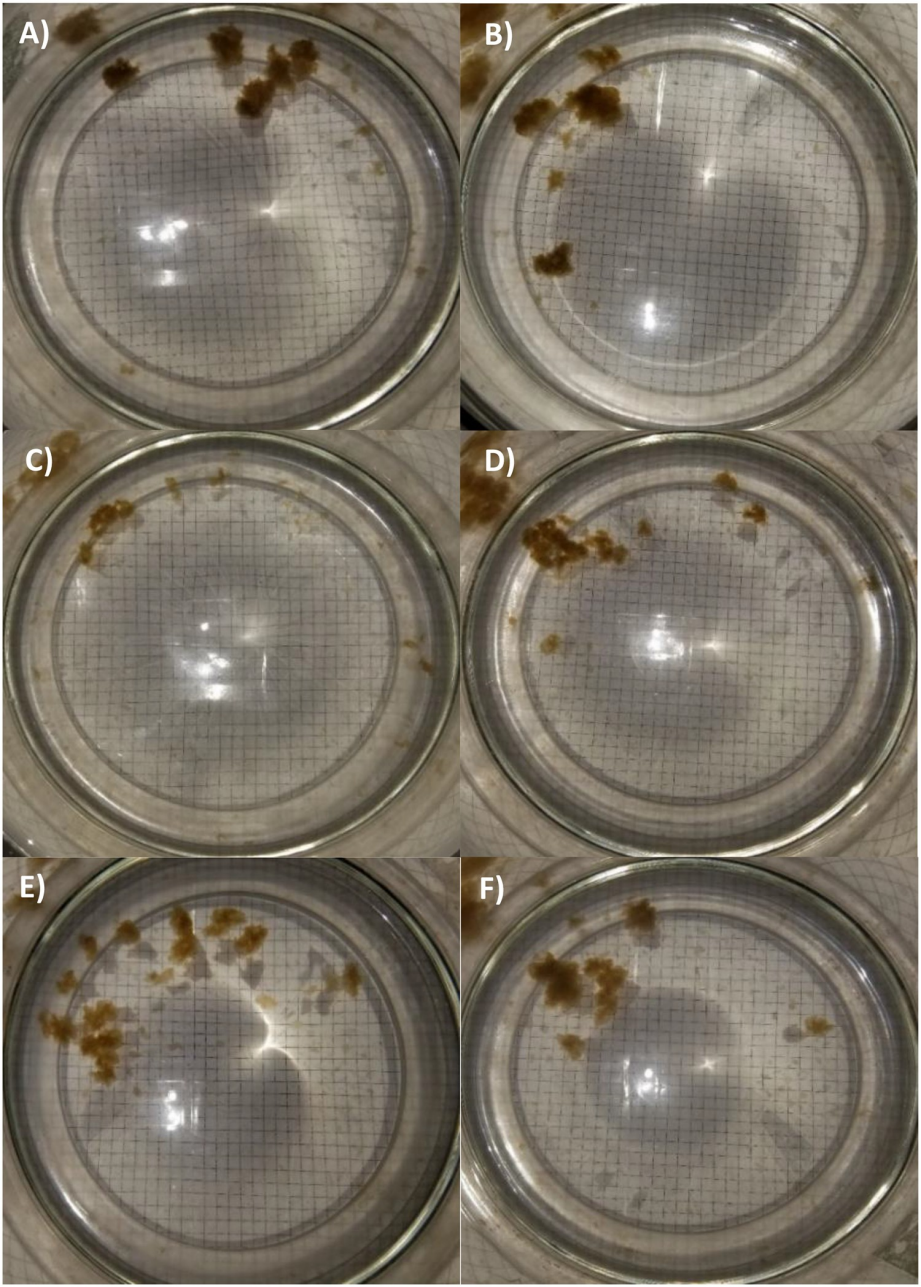
Photographs of marine snow formed by *T*. *pseudonana* in each treatment: (A) Control, (B) OA, (C) WAF, (D) OAWAF, (E) DCEWAF and (F) OADCEWAF. Grid squares 0.5 cm by 0.5 cm.

Images of aggregates in each of the six treatments show the variability in size, shape, and number among treatments (Fig 1A–1F). Visible aggregates formed in Control and OA within three hrs, DCEWAF and OADCEWAF treatments after 24 hrs, and in WAF and OAWAF after 60 hrs [55]. Image analysis of the aggregates determined the variation in counts and sizes with treatment for *T*. *pseudonana* (Table 2). Controls had the fewest total counts (53.67 ± 2.31 aggregates), while OADCEWAF tanks had the most per replicate (100.67 ± 67.88 aggregates). There were no significant differences between treatments (p = 0.4549, F = 1.00648). There were also no Large (>4.683 cm^2^) aggregates in WAF tanks, but they were present in OAWAF (Table 2; Fig 1). Of the Large aggregates, those with the greatest area were present in the DCEWAF (7.64 ± 2.73 cm^2^), OA (6.84 ± 2.20 cm^2^), OADCEWAF (6.49 ± 1.37 cm^2^) and those with the least in the Control (5.37 cm^2^) OAWAF (4.68 cm^2^) (Table 2). Although distribution among size classes changed due to *p*CO_2_ levels, the total aggregate was not significantly (p = 0.06418, F = 2.84173) affected by OA (Table 2; Fig 1). Instead, total aggregate area was driven by the presence of oil and oil plus dispersant. The total aggregate area was lowest in the WAF and OAWAF treatments (3.46 ± 0.80 cm^2^ and 4.58 ± 2.96 cm^2^, respectively), followed by the Controls/OA (5.19 ± 3.40 cm^2^ and 10.04 ± 2.40 cm^2^, respectively), and highest in the DCEWAF/OADCEWAF (8.96 ± 5.44 cm^2^ and 10.26 ± 1.04 cm^2^, respectively) (Table 2; Fig 1).

**Table 2:** Aggregate counts and area (cm^2^) by size class in the roller table experiments. Error bars in brackets are standard deviation of triplicates.

	Counts						Area (cm^2^)					
Size Class	Control	OA	WAF	OAWAF	DCEWAF	OADCEWAF	Control	OA	WAF	OAWAF	DCEWAF	OADCEWAF
**Large** (>4.683)	0.33 (±0.47)	1 (± 0)	0	0.33 (±0.47)	0.67 (±0.47)	1.67 (±0.47)	5.37 (±0)	6.84 (±2.20)	0	4.68 (±0)	7.64 (±2.73)	6.49 (±1.37)
**Medium** (1.551–4.002)	4 (±1.41)	3 (±1.63)	1 (±0)	1.67 (±0.47)	4.33 (±1.70)	2 (±0.82)	2.62 (±0.03)	2.32 (±0.39)	2.50 (±0.70)	2.04 (±0.30)	2.70 (±0.12)	2.60 (±0.74)
**Small** (0.591–1.464)	4.33 (±3.30)	3.33 (±2.62)	6 (±2.16)	6.67 (±3.68)	4.67 (±1.70)	3 (±2.16)	1.02 (±0.01)	0.82 (±0.11)	0.84 (±0.10)	0.85 (±0.07)	1.06 (±0.11)	1.04 (±0.21)
**XS** (0–0.572)	45 (±4.08)	52.67 (±8.06)	68.67 (±13.3)	58.67 (±9.81)	48.67 (±4.11)	94 (±56.7)	0.09 (±0.01)	0.07 (±0.01)	0.12 (±0.01)	0.12 (±0.02)	0.10 (±0.01)	0.13 (±0.02)
**Total**	53.67 (±2.31)	60 (±14.9)	75.67 (±18.9)	67.33 (±7.5)	58.33 (±6.43)	100.67 (±67.88)	5.19 (±3.40)	10.04 (±2.40)	3.46 (±0.80)	4.58 (±2.96)	8.96 (±5.44)	10.26 (±1.04)

**Fig 2:**
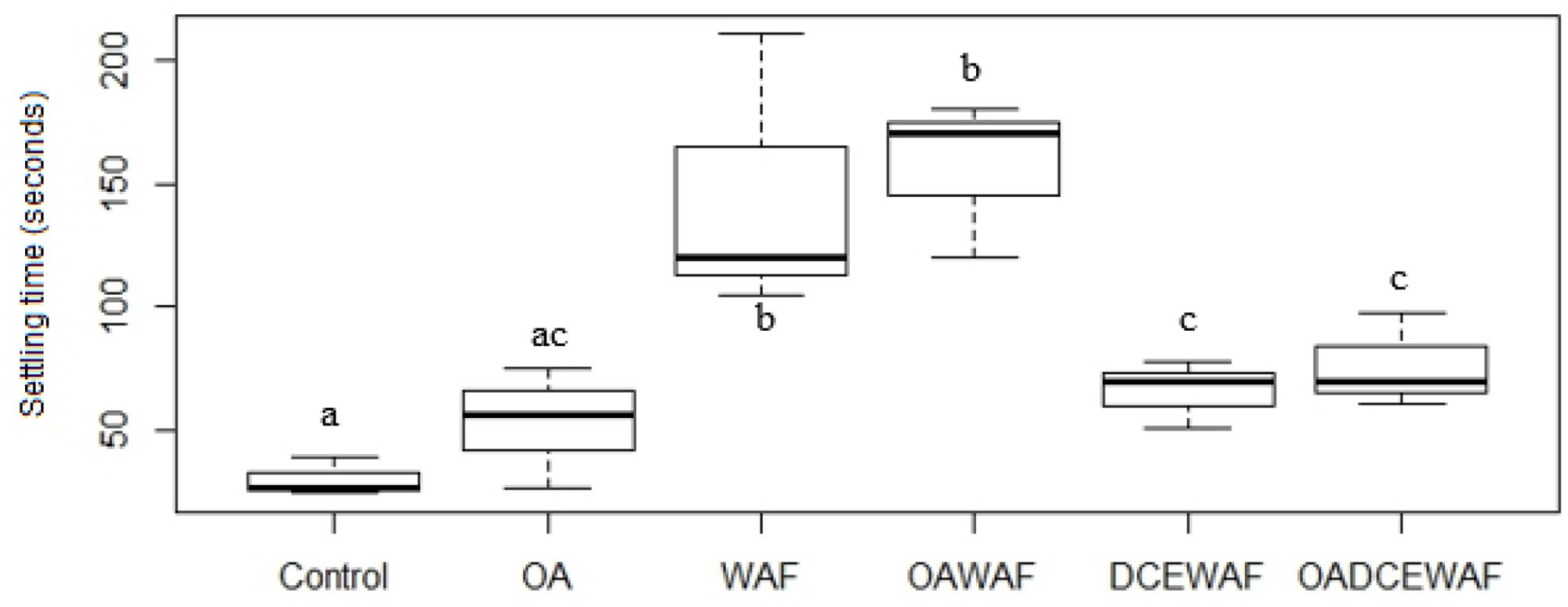
Settling time (sec) for all aggregates of *T*. *pseudonana* at the end of the roller table experiments. Box and whisker plots with mean values (dark bar) and error bars of the maximum and minimum values for replicates. Different letters indicate which treatments are significantly (*p*<0.05) different from each other.

The settling times for aggregates in each roller tank were recorded at the end of the incubation period (Fig 2). Control aggregates settled the fastest (30.6 ± 8.1 sec), followed by OA (53 ± 24.24 sec), DCEWAF (66.33 ± 13.86 sec), OADCEWAF (76.33 ± 19.29 sec), WAF (145 ± 56.78 sec), and OAWAF (156.67 ± 32.14 sec). The differences across treatments were significantly different (Two-way ANOVA, p = 0.00116, DF = 5, F = 8.598). Both WAF and OAWAF treatments had remaining suspended aggregate formations after 2 mins.

**Fig 3:**
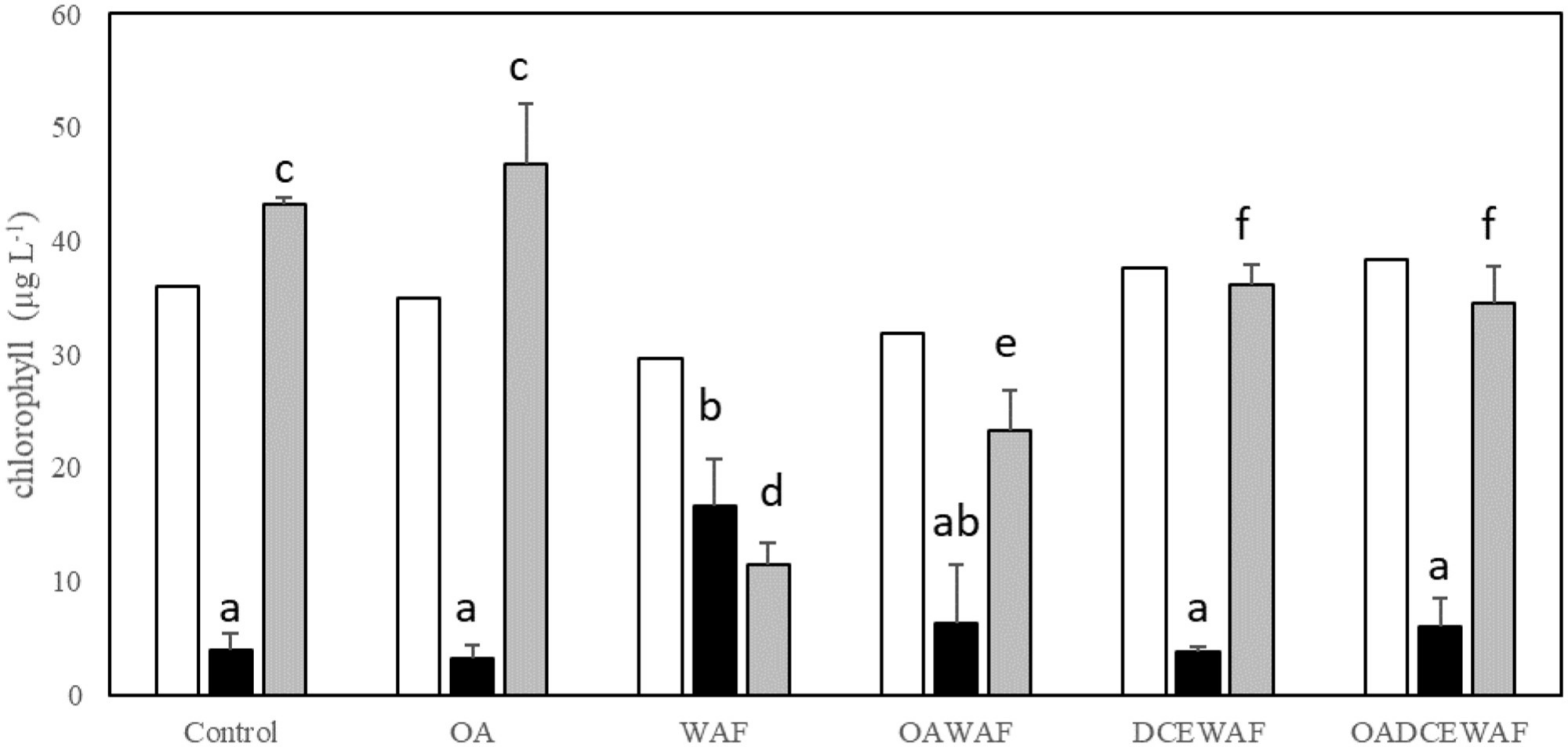
Chl *a* biomass (ug L^-1^) of *T*. *pseudonana* in the SSW at the start (white) and the end (black) of the roller table experiments, as well as the Chl *a* biomass in the AGG (grey bars). Error bars show standard deviation of triplicates. Different letters indicate which treatments are significantly (*p*<0.05) different from each other.

Chl *a* measurements were taken for the beginning SSW, and the SSW and AGG fractions collected at the end of the exponential growth phase experiment (Fig 3). The majority of *T*. *pseudonana* cells moved into the aggregates in the Control (91%) and OA (93%) treatments, less so in the DCEWAF and OADCEWAF (90% and 85%), and least of all in the WAF and OAWAF treatments, 40% and 78% respectively. The effect of elevated *p*CO_2_ on AGGs (as chl *a* biomass) was negligible between Control and OA (p = 0.4454) and DCEWAF and OADCEWAF (p = 0.5922), but there was a significant difference between WAF and OAWAF treatments (p = 0.02487). There was a significant accumulation into the aggregate material in the DCEWAF and OADCEWAF compared to Control or OA (p = 0.0233 and p = 0.06448, respectively). Overall, most chl *a* biomass was present in WAF/OAWAF aggregates (p = 0.000819).

### Extracellular materials produced by *T*. *pseudonana*

**Fig 4:**
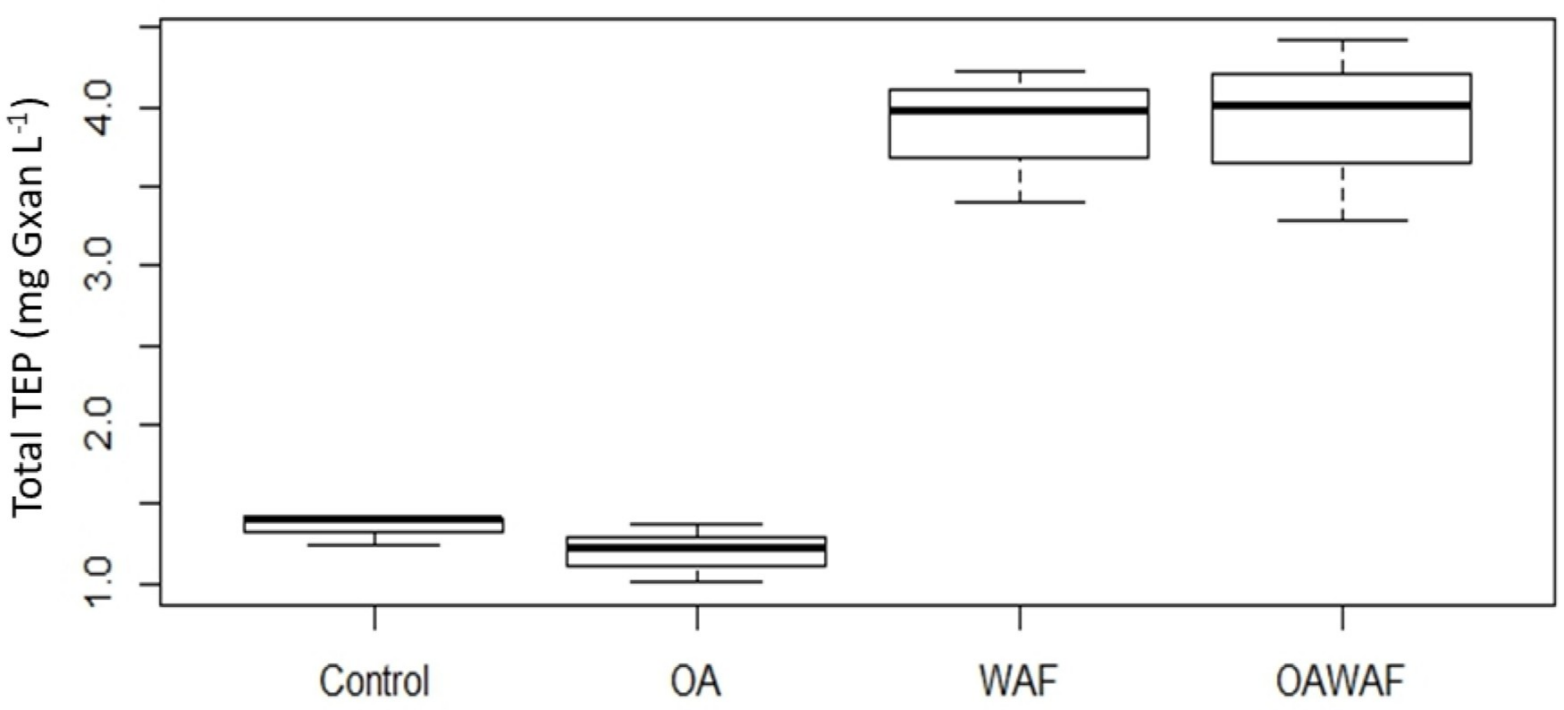
TEP measured at the end of the roller table experiments in the SSW only for Control, OA, WAF, and OAWAF. DCEWAF and OADCEWAF could not be measured (see Methods for technical details). Box and whisker plots with mean values (dark bar) and error bars of the maximum and minimum values for replicates. Different letters indicate which treatments are significantly (*p*<0.05) different from each other.

**Fig 5:**
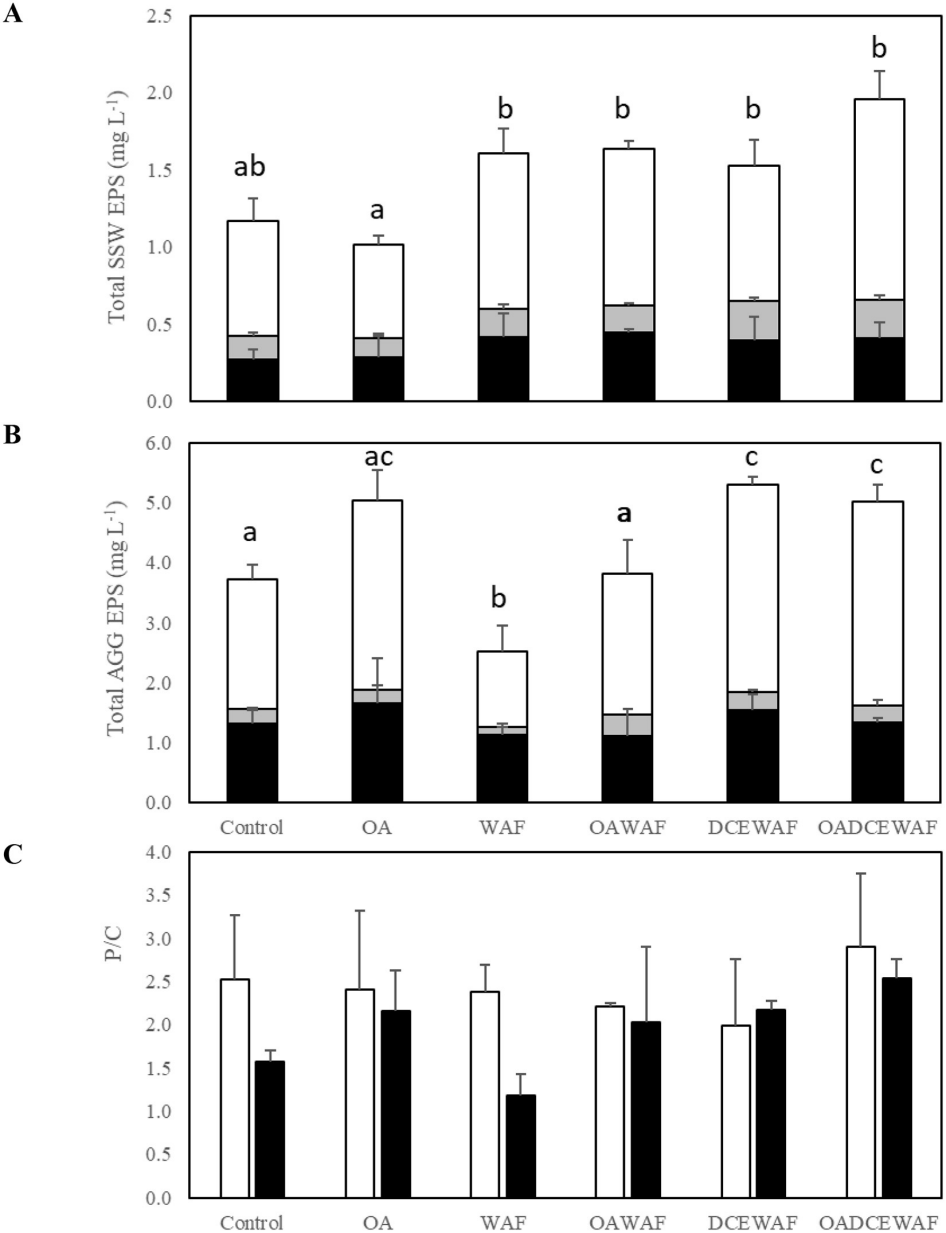
EPS was measured in cultures of *T*. *pseudonana* at the end of the roller table experiments. Neutral sugars (black), uronic acids (grey), and proteins (white) are stacked to show the **(A)** total colloidal EPS concentration in the surround seawater (SSW) and the **(B)** particulate EPS concentration in the AGGs. **(C)** Protein/carbohydrate (P/C) ratios of EPS in the SSW (white) and AGGs (black). Error bars show standard deviation of triplicates. Different letters indicate which treatments are significantly (*p*<0.05) different from each other.

It was observed that WAF and OAWAF treatments led to the production of similar TEP concentrations in the SSW (Fig 4), but produced significantly (p<0.05) more than in either the Control or OA treatments. TEP was not measured in DCEWAF or OADCEWAF as Corexit interferes with the protocol [58]. The colloidal EPS (<0.7 μm to >3 kDa) of the SSW (Fig 5A) and the particulate EPS of the AGG (Fig 5B) was measured as the sum of proteins, neutral sugars and uronic acids. Proteins dominated the colloidal EPS in all treatments compared to the total carbohydrates (neutral sugars + uronic acids). The colloidal EPS was not significantly different (p>0.05) between oil and oil plus dispersant treatments, with WAF (1.60 ± 0.34 mg L^-1^), OAWAF (1.63 ± 0.09 mg L^-1^), DCEWAF (1.52 ± 0.35 mg L^-1^) and OADCEWAF (1.96 ± 0.32 mg L^-1^), all producing similar amounts (Fig 5A). However, there was significantly less colloidal EPS in Control (1.17 ± 0.23 mg L^-1^) and OA (1.01 ± 0.23 mg L^-1^) treatments. The P/C ratio for the colloidal EPS did not vary significantly between any of the treatments (p>0.05), ranging from 2.0 to 2.9 (Fig 5C).

Proteins also dominated the particulate EPS fraction relative to carbohydrates (Fig 5B). *T*. *pseudonana* produced significantly (p<0.05) more particulate EPS in the DCEWAF (5.30 ± 0.40 mg L^-1^) and OADCEWAF (5.02 ± 0.44 mg L^-1^) treatments than the Control (3.73 ± 0.45 mg L^-1^) and WAF (2.53 ± 0.57 mg L^-1^) treatments (and the corresponding OA and OAWAF treatments). The AGG P/C ratio in the particulate EPS was significantly higher in DCEWAF (2.17 ± 0.11) and OADCEWAF (2.54 ± 0.21) than in Control (1.57 ± 0.13) (p<0.01), but was not significantly different between any of the other treatments (Fig 5C). The lowest particulate EPS P/C ratio was measured in WAF (1.18 ± 0.25). For the particulate EPS, it appeared that the P/C ratios were always lower in the Control/WAF/DCEWAF than the corresponding OA treatments, but the finding was not statistically significant.

### Oil analysis

The EOE concentrations were similar between treatments at the start of the roller table experiment and ranged between 0.2 to 0.26 mg L^-1^ in WAF, OAWAF, DCEWAF and OADCEWAF (Table 3). The final EOE concentrations were more variable, ranging from 0.08 to 0.18 mg L^-1^. Oil concentrations (EOE and PAH) were the under detection limit for the Control and OA treatments.

**Table 3:** Descriptions of the treatments used for the experiments. Initial (one sample) and final (triplicate) EOE (mg L^-1^) and initial PAH (ng L^-1^) and PAH (μg L^-1^) in SSW and AGG measurements are included. Error bars in brackets are standard deviation of triplicates.

Treatment	Control	OA	WAF	OAWAF	DCEWAF	OADCEWAF
Inoculation volumes	5.3 L ASW 0.351 L culture	5.3 L ASW 0.32 L culture	5.4 L WAF 0.27 L culture	5.3 L WAF 0.32 L culture	5.2 L ASW 0.001 L CEWAF 0.45 L culture	5.2 L ASW 0.001 L CEWAF 0.43 L culture
Initial EOE	0	0	0.26	0.2	0.2	0.2
EOE (SSW)	0	0	0.13 (± 0.01)	0.11 (± 0.01)	0.18 (± 0.12)	0.08 (± 0.02)
Initial PAH	0.15	0.09	61.27	60.29	2.13	2.56
PAH (SSW)	0.41 (± 0.04)	0.21 (± 0.01)	41.12 (± 6.02)	37.02 (± 3.45)	2.08 (± 0.16)	2.18 (± 0.16)
PAH (AGG)	3.38 (± 2.05)	0.80 (± 0.35)	145.73 (± 76.85)	155 (± 44.16)	77.54 (± 13.76)	69.04 (± 6.40)

Initial PAH concentrations were higher in WAF and OAWAF (60.29 ng L^-1^ and 61.27 ng L^-1^, respectively) than in DCEWAF and OADCEWAF (2.56 ng L^-1^ and 2.75 ng L^-1^, respectively). The concentrations of PAHs incorporated into AGG at the end of the roller table experiments in the WAF (145.73 ± 76.85 μg L^-1^) and OAWAF (155 ± 44.16 μg L^-1^) treatments were higher than in the DCEWAF (77.54 ± 13.56 μg L^-1^) or OADCEWAF (69.04 ± 6.40 μg L^-1^) (p = 0.02242, F = 7.27566) (Table 3).

**Fig 6:**
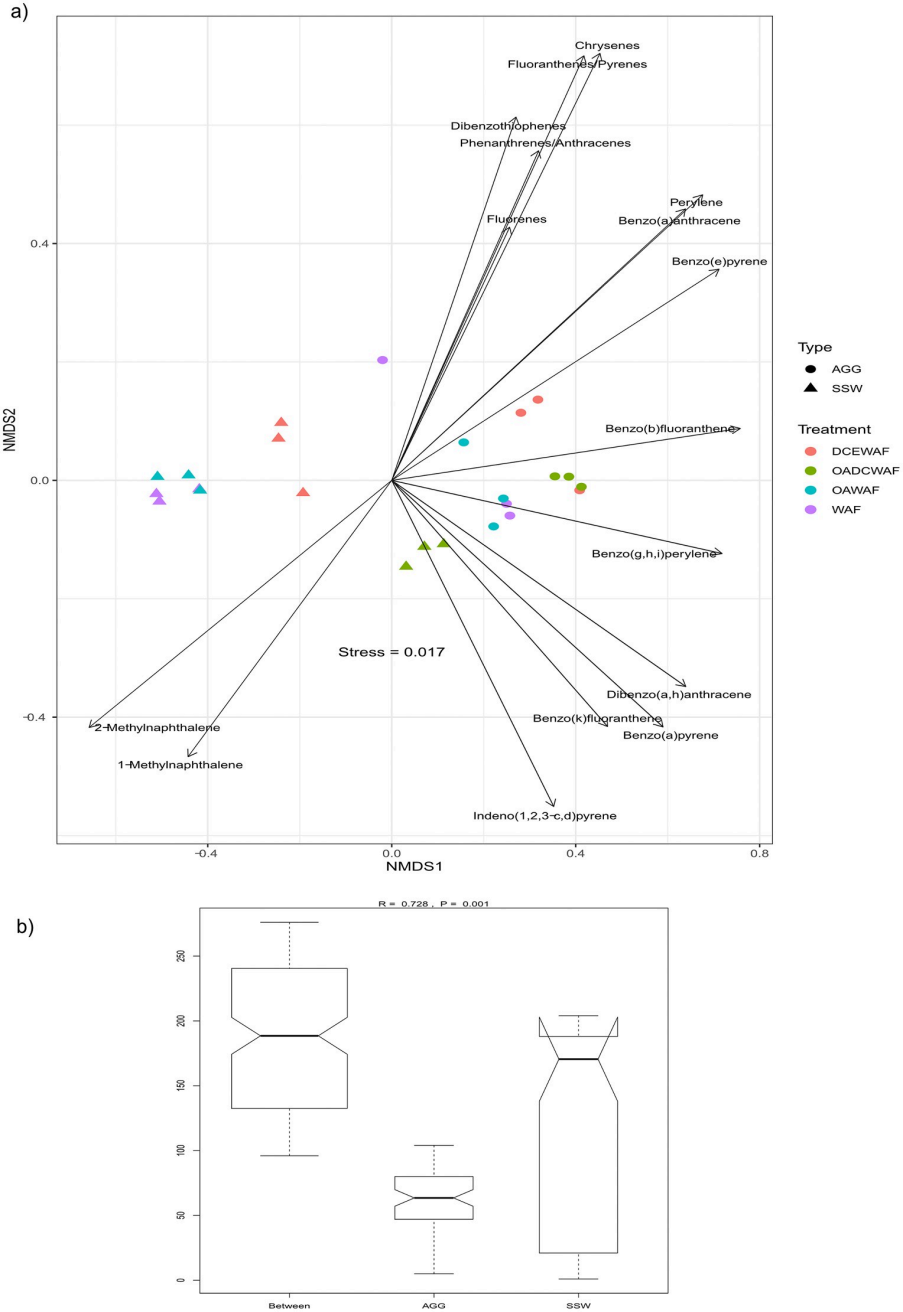
Analysis of PAH composition in AGG and SSW under all the treatments excluding control and OA using A) NMDS and B) ANOSIM.

Differences in PAHs composition between AGG and SSW in every treatment are provided in S1 Table. These were visualized in an NMDS plot (Fig 6; S1 Fig). The near absence of PAH components in Control and OA treatments led to a clear separation between Control AGG and SSW from all other treatments, making it difficult to assess the differences between AGG and SSW of WAF, OAWAF, DCEWAF and OADCEWAF. Therefore, the NMDS plot was recreated without Control AGG and SSW (Fig 6A). A clear separation between AGG and SSW was observed for all WAF, OAWAF, DCEWAF and OADCEWAF treatments. In addition, differences in clustering was observed for WAF, OAWAF vs DCEWAF and OADCEWAF in both AGG and SSW fraction. Except for the AGG fraction of DCEWAF and OADCEWAF, there were no differences in clustering observed between AGG and SSW of all the treatments. Analysis of Similarities (ANOSIM) suggested that the separation observed in NMDS plot between AGG and SSW fraction was significant (p = 0.001, R = 0.728) (Fig 6B). However, the dissimilarities between OA and non-OA treatments were low compared to the difference between oil (WAF and OAWAF) and oil plus dispersant (DCEWAF and OADCEWAF) treatments (p = 0.001, R = 0.4485) (Fig 5B, S2a Fig).

The PAH components with significant differences were further investigated through SIMPER analysis. PAH composition were 60.58% different between AGG and SSW, with components such as Phenanthrenes and Anthracenes contributing the most to the overall dissimilarity (p = 0.001), followed by Chrysenes, Fluorenes, Fluoranthenes, Pyrenes, Dibenzothiophenes and 1-Methylphenanthrene (p = 0.001) (S1 and S2 Tables). The DCEWAF/OADCEWAF treatments were 52.76% different from WAF/OAWAF treatments in PAH composition, with components such as Naphthalenes contributing the most to the overall dissimilarity (p = 0.001), followed by 1-Methylnaphthalene, Fluorenes, 2-Methylnaphthalene and 1-Methylphenanthrene (S3 Table).

The ANOSIM showed that AGG PAH composition was significantly different between WAF and DCEWAF (p = 0.003, R = 0.848) (S2 Fig). SIMPER indicated that DCEWAF AGG was ~25% different from WAF, and that Napthalenes and Fluorenes contributed the most to overall observed dissimilarities (p = 0.004) (S2 Table). Similarly, the PAH composition of the SSW was significantly different between WAF and DCEWAF (p = 0.003, R = 1) (S2 Fig). Naphthalenes and 2-Methylnaphthalene contributed the most to the observed overall dissimilarities (p = 0.002), followed by 1-Methylnaphthalene, Phenanthrenes, Anthracenes, 2,6-Dimethylnaphthalene, Biphenyls and Fluorenes (p<0.005) (S2 Table and Fig 2).

## Discussion and conclusions

After the DwH oil spill, several studies identified diatoms as major responders to the event and contributors towards the development of MOS and MOSSFA [14, 15, 58]. Previous laboratory studies, which examined the response of individual phytoplankton strains to oil and chemically dispersed oil, have shown *T*. *pseudonana* to be a sensitive species with reduced growth rates and lowered photosynthetic activity [12, 13]. Additionally, studies of this diatoms’ response to OA have found a variety of physiological responses even when the same strain was used [25, 38, 39, 40, 41]. The results of the present investigation indicated that *T*. *pseudonana*’s response, after acclimation to current and future ocean *p*CO_2_, was primarily due to the addition of WAF or DCEWAF, and not the OA conditions. That is, there was not a multiplicative effect of both factors on this diatom. We did, however, find differences between exudate production and aggregation formation by *T*. *pseudonana* between Controls, WAF and DCEWAF (Fig 1). For the latter, similar observations have been reported previously [58, 63]. A negative effect from the addition of Corexit on aggregate formation was also observed in a mesocosm study with nearshore surface water [11, 62], other roller table studies [58] and bottle experiments [64]. Also of great significance is that there were no differences which could be directly associated to OA. This was a consistent result, whether aggregates were visually compared (Fig 1 and Table 3), or chemically analyzed (Figs 2–5). As a general observation, we observed aggregates in the “Large” size class range in the OA treatments. We hypothesize this was driven by the seawater chemistry on aggregate formation rather than a physiological response of *T*. *pseudonana*. This is also consistent with those previous publications that found *T*. *pseudonana* did not respond to OA conditions [25, 38, 39]. There is a detailed literature which has endeavored to address why a response of *T*. *pseudonana* was observed in some studies [40, 41] to which the reader is directed.

EPS is integral to the formation of aggregates in the ocean [5, 6]. EPS and TEP are amphiphilic macromolecules released into the environment by microbial organisms and are essential for aggregation and the biological carbon pump [17]. The amphiphilic nature of these molecules is similar to chemical dispersants, which decrease the surface tension of water and can enhance interactions with oil [5, 65]. In order to produce this aggregate material, *T*. *pseudonana* released exudates composed primarily of proteins and carbohydrates (polysaccharides). Operational definitions for TEP and EPS have been defined based on established protocols [5, 66]. TEP protocols measure the polysaccharides released by phytoplankton [17, 27, 30, 36]. Previous studies have found that TEP production increases in higher *p*CO_2_ when examining natural phytoplankton communities [67, 68] or the coccolithophore *Emiliania huxleyi* [69, 70], but not amongst diatoms (e.g., *Thalassiosira weissflogii* [23], *Chaetoceros muelleri* [71]. *T*. *pseudonana*). TEP production was not influenced by OA in the current study, but *T*. *pseudonana* did produce more TEP in the WAF treatments than in the corresponding Controls. These organic materials may even be more efficient than synthetic surfactants at dispersing oil by forming micelles [65], and have been shown to emulsify and incorporate oil into EPS, TEP and/or marine snow [11, 63, 67].

In this study, colloidal EPS was measured in SSW, while particulate EPS was measured in AGG materials. In both fractions, there was a common trend of total material produced: Controls < WAF < DCEWAF. Even though there was more EPS produced in the DCEWAF/OADCEWAF treatments relative to the Control/OA, more biomass remained suspended in the SSW in the DCEWAF/OADCEWAF treatments. This is consistent with observations from image analysis of denser aggregates present in Controls relative to DCEWAF, as well as previous studies [55, 66]. The area of the aggregate material was also measured as this is known to influence sinking rates. When comparing settling times between treatments, the aggregate material in the WAF/OAWAF treatments were ultimately slower than in Control/OA and DCEWAF/OADCEWAF. This may be a reflection of how much oil was associated with individual aggregates and the role of dispersant in modifying the interaction between the oil and aggregate material [58, 72,73]. Alternatively it maybe because the Control/OA and DCEWAF/OADCEWAF have a higher proportion of dead cells (more diatom shells but less living material) in the aggregate than WAF/OAWAF, which could then also partially explain the shorter settling time of the previous two sets of treatments than that of the WAF/OAWAF treatments. While it is known that oil is less dense than water, thus potentially increasing the buoyancy of aggregates [11], the inclusion of oil also affects the packaging of aggregates, which would alter the composition of the MOS and increase sinking rates and density [4, 58, 63, 72, 73].

The EPS P/C ratio has been determined as a proxy for stickiness and is associated with lower surface tension [29, 62, 65]. The P/C ratios for the colloidal EPS were generally higher (2.0–2.9) than those in the particulate EPS (1.18–2.54), suggesting the material released by the cells was stickier than that associated with the cells. These have previously been referred to as “unattached” versus “attached” EPS [11, 62] and typically have different P/C ratios depending on the phase of the cells growth cycle and the stresses upon the cells [29, 74, 75, 76]. Again, there were no significant differences due to OA; only the oil and oil plus dispersant. WAF produced EPS with lower P/C ratio compared to control; CEWAF or DCEWAG produced EPS with higher P/C ratios. Similar observations were made during mesocosm experiments with natural communities [11, 62] dominated by diatoms, and thus the findings may be reflective of a more general diatom response [11, 12, 13, 66]. The TEP and EPS findings build on previous studies that found the presence of oil (WAF) and oil plus dispersant (DCEWAF) led to an increase in exudate release and aggregation of *T*. *pseudonana* [12]. The P/C ratio for *T*. *pseudonana* EPS is higher than in other phytoplankton, and therefore this exudate is more hydrophobic in nature [75, 77]. This hydrophobicity may explain why the aggregates were not dispersed by the Corexit in DCEWAF, and why EPS aggregation state was elevated [77, 78]. The WAF aggregates had the lowest ratio, meaning they were the least sticky, and had the least amount of total aggregate area produced in the experiment. These results promote and give new insights of the importance of proteins for marine snow formation. While some earlier studies of Coomassie stained particles, or protein exudates, found these proteins were not essential for aggregation [79], recent studies measuring both proteins and carbohydrates have reported they indeed play a key role [29].

We found that there was much more PAH in the *T*. *pseudonana* DCEWAF aggregates than in the WAF treatments (Table 2). PAHs were of particular interest because they are known to last in the water column for years after a spill and are some of the most toxic components of crude oil to marine organisms [14, 80, 81, 82]. Passow et al. [58] developed a conceptual diagram to explore the complex interactions between the aggregate material, oil and dispersant. Our results show that OA had very minimal effects on the PAH composition between AGG and SSW in the presence or absence of dispersants (Fig 6). However, we found that dispersants significantly affected the PAH composition in AGG and SSW fractions. Several studies [11, 58, 72] have found that the addition of Corexit dispersant affected the oil compounds accumulating within MOS. We found that PAHs such as Napthalenes, 1-Methylnaphthalene, Fluorenes, 2-Methylnaphthalene and 1-Methylphenanthrene were differentially present in treatments with and without dispersant. These observations were similar if not identical to those found by Bacosa et al. [73]. Passow et al. [63] also showed that aggregates vary in their carrying capacity for oil compounds compared to the surrounding seawater. In agreement with this observation, we found that the PAH composition of AGG and SSW were significantly different, with PAHs such as Phenanthrenes, Anthracenes, Chrysenes, Fluorenes, Fluoranthenes, Pyrenes, Dibenzothiophenes and 1-Methylphenanthrene showing major variations. Overall, we found that OA had very minimal effects on the PAH composition between AGG and SSW in the presence or absence of dispersants. However, we found that dispersants significantly affected the PAH composition in AGG and SSW fractions.

The results from our studies are not only important for understanding interactions of oil/dispersant/OA at the species level, but also for envisioning the potential of overall effects to the ecosystem. The majority of organic carbon in the ocean is non-living, so if changes to pH impact the aggregation and sinking of this material, then it may ultimately lead to changes in aggregate sinking rate. Aggregates contribute a significant amount of carbon to the seafloor [43], and sink faster than diatom cells alone [26, 28, 43], with several studies proposing that OA will lead to increased aggregate formation and carbon flux [25, 34, 35, 36]. With increasing oil exploration activities, oil spills will only become more common in the future, therefore exposing the primary producers of ocean to multiple layers of threat. Our study shows that although factors such as OA and oil spill have the potential to alter phytoplankton physiology, the use of dispersants further alters the behavior of diatom led aggregation process, but there was no compounding effects of these factors.

## Supporting information

S1 Table PAH composition and concentration in AGG and SSW fractions in both control and pCO2 conditions of all the treatments. (DOCX)

S2 Table Summary of SIMPER analysis of PAH composition from AGG and SSW fraction. (DOCX)

S3 Table Summary of SIMPER analysis of PAH composition from OA (OAWAF and OADCEWAF) and non-OA (WAF and DCEWAF) treatments. (DOCX)

S1 Fig Analysis of PAH composition in AGG and SSW under all the treatments using NMDS plot. (TIFF)

S2 Fig A Analysis of dissimilarities in PAH composition in AGG and SSW under all the treatments using ANOSIM B & C Analysis of PAH composition in AGG and SSW (WAF and OAWAF) and oil plus dispersant (DCEWAF and OADCEWAF) treatments using ANOSIM, respectively. (TIFF)
