# Supplementary material for: Diatom aggregation when exposed to crude oil and chemical dispersant: Potential impacts of ocean acidification
Source: PLoS One. 2020 Jul 7;15(7):e0235473. doi: 10.1371/journal.pone.0235473 (PMC7340286; doi:10.1371/journal.pone.0235473)
Supplement: S3 Table — (DOCX) [file pone.0235473.s003.docx]

| **PAH in DCEWAF vs OADCEWAF** | **average** | **sd** | **ratio** | **ava** | **avb** | **cumsum** | **p** |  |
| --- | --- | --- | --- | --- | --- | --- | --- | --- |
| Phenanthrenes/Anthracenes | 0.071046 | 0.066033 | 1.076 | 95.209 | 89.205 | 0.1856 | 0.249 |  |
| Chrysenes | 0.053496 | 0.049226 | 1.087 | 61.133 | 60.569 | 0.3254 | 0.018 | * |
| Fluoranthenes/Pyrenes | 0.040584 | 0.0362 | 1.121 | 49.284 | 46.574 | 0.4314 | 0.022 | * |
| Naphthalenes | 0.034032 | 0.030005 | 1.134 | 70.466 | 69.394 | 0.5203 | 1 |  |
| Dibenzothiophenes | 0.032742 | 0.027557 | 1.188 | 43.606 | 40.682 | 0.6059 | 0.142 |  |
| Fluorenes | 0.031788 | 0.028792 | 1.104 | 48.461 | 45.126 | 0.6889 | 0.913 |  |
| 1-Methylphenanthrene | 0.013778 | 0.012683 | 1.086 | 20.431 | 18.885 | 0.7249 | 0.96 |  |
| Benzo(e)pyrene | 0.011786 | 0.00693 | 1.701 | 10.019 | 12.075 | 0.7557 | 0.005 | ** |
| 2,6-Dimethylnaphthalene | 0.011083 | 0.010633 | 1.042 | 22.154 | 19.379 | 0.7847 | 0.797 |  |
| Perylene | 0.010226 | 0.009297 | 1.1 | 11.436 | 11.39 | 0.8114 | 0.029 | * |
| 1,6,7-Trimethylnaphthalene | 0.008529 | 0.007567 | 1.127 | 15.362 | 14.838 | 0.8337 | 0.998 |  |
| Benzo(b)fluoranthene | 0.008425 | 0.007345 | 1.147 | 8.911 | 9.023 | 0.8557 | 0.044 | * |
| Benzo(a)anthracene | 0.007418 | 0.006621 | 1.12 | 8.299 | 8.77 | 0.8751 | 0.03 | * |
| Benzo(g,h,i)perylene | 0.006552 | 0.004804 | 1.364 | 5.99 | 6.715 | 0.8922 | 0.026 | * |
| 2-Methylnaphthalene | 0.006378 | 0.004558 | 1.399 | 20.725 | 20.711 | 0.9088 | 0.998 |  |
| Dibenzo(a,h)anthracene | 0.005618 | 0.003848 | 1.46 | 4.392 | 5.826 | 0.9235 | 0.018 | * |
| 1-Methylnaphthalene | 0.005494 | 0.003727 | 1.474 | 17.512 | 17.934 | 0.9379 | 1 |  |
| Benzo(a)pyrene | 0.005106 | 0.003691 | 1.383 | 4.157 | 5.387 | 0.9512 | 0.017 | * |
| Indeno(1,2,3-c,d)pyrene | 0.004743 | 0.003485 | 1.361 | 2.191 | 4.844 | 0.9636 | 0.023 | * |
| Benzo(k)fluoranthene | 0.004558 | 0.004146 | 1.099 | 3.551 | 5.379 | 0.9755 | 0.066 | . |
| Biphenyl | 0.004544 | 0.003616 | 1.257 | 10.345 | 10.499 | 0.9874 | 1 |  |
| Acenaphthylene | 0.002794 | 0.00241 | 1.16 | 4.643 | 3.798 | 0.9947 | 0.851 |  |
| Acenaphthene | 0.002038 | 0.001558 | 1.308 | 3.413 | 3.768 | 1 | 0.931 |  |
