# Supplementary figures and images for: Diatom aggregation when exposed to crude oil and chemical dispersant: Potential impacts of ocean acidification
Source: PLoS One. 2020 Jul 7;15(7):e0235473. doi: 10.1371/journal.pone.0235473 (PMC7340286; doi:10.1371/journal.pone.0235473)

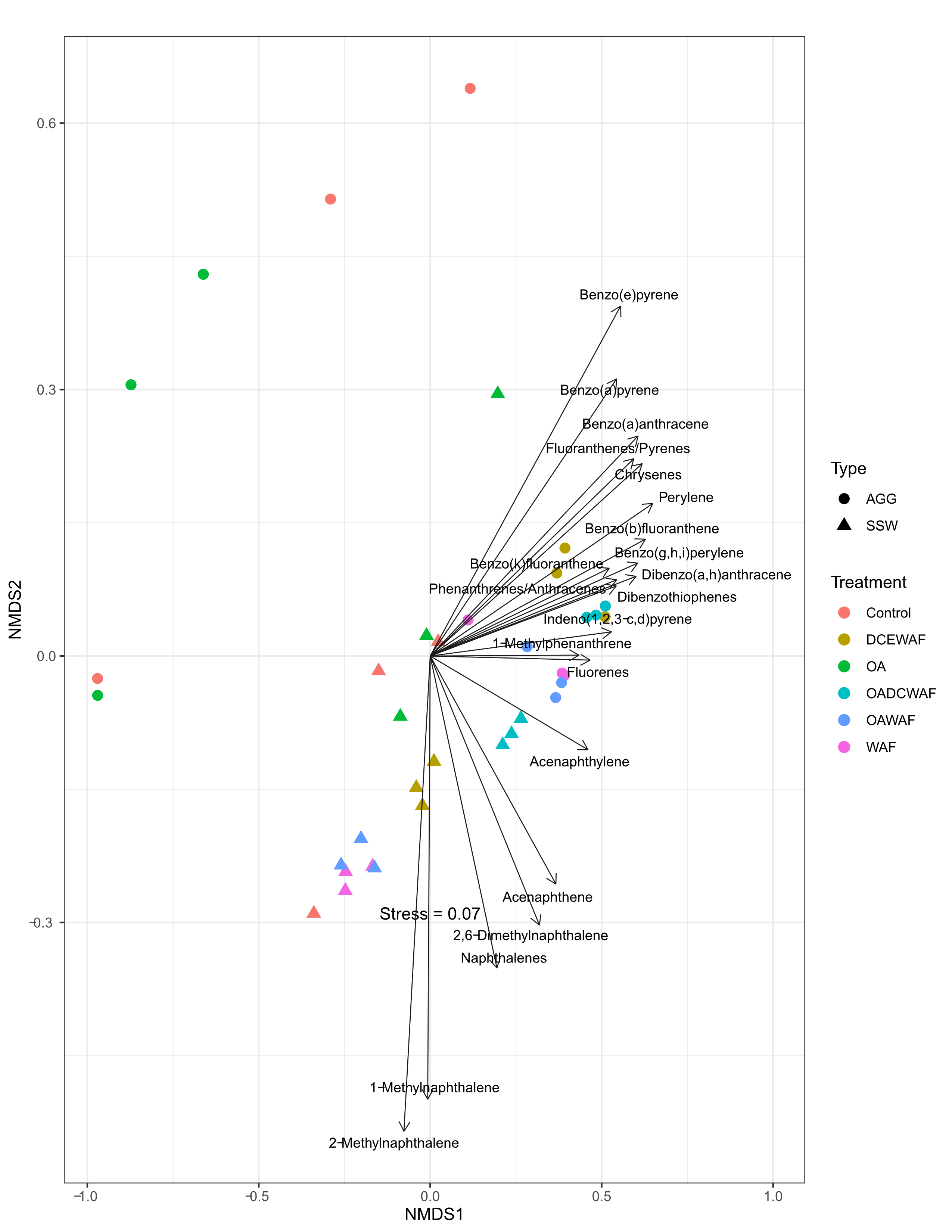

Supplement: S1 Fig — (TIFF) [file pone.0235473.s004.tiff]

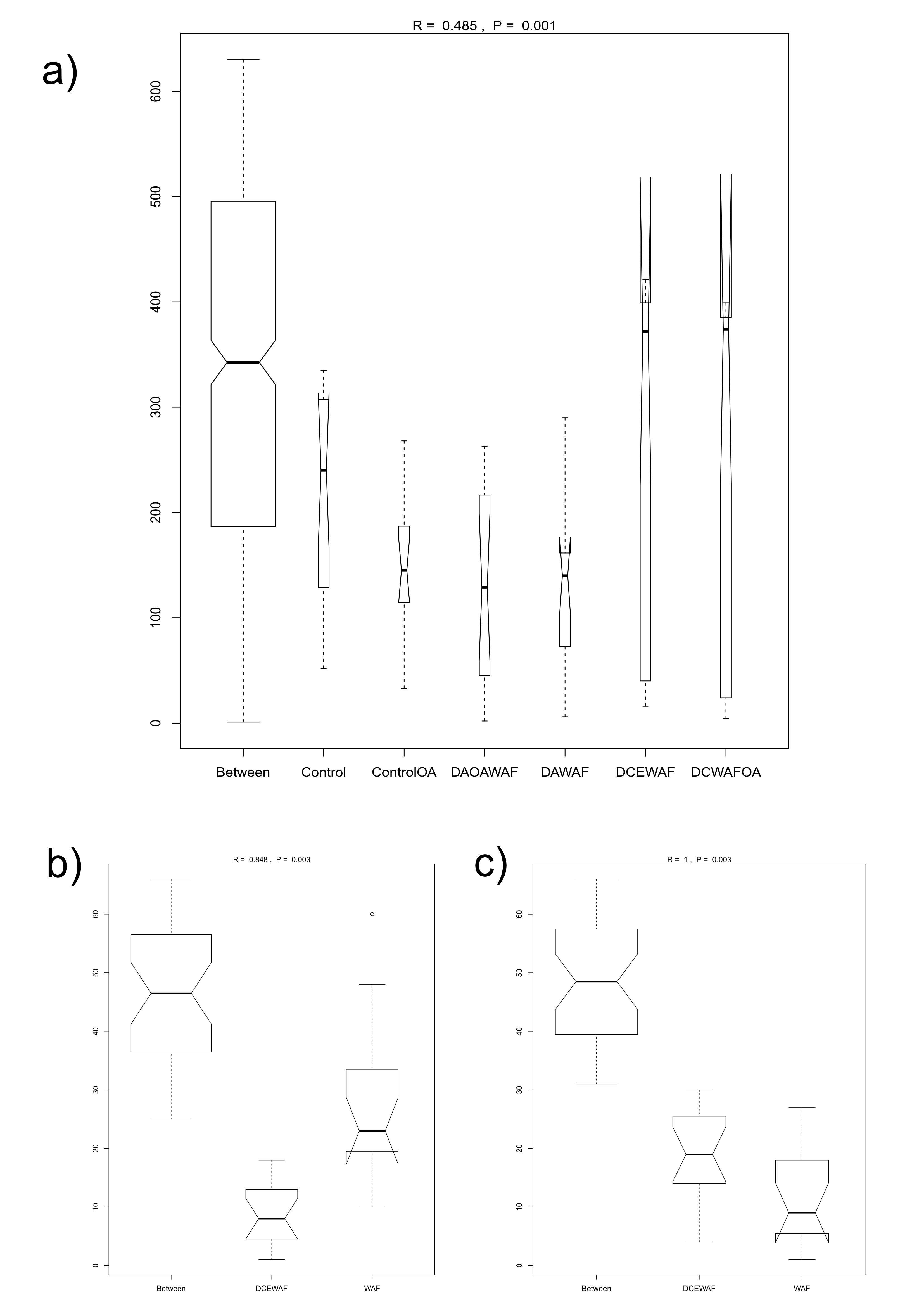

Supplement: S2 Fig — (TIFF) [file pone.0235473.s005.tiff]
