# Supplementary material for: Diatom aggregation when exposed to crude oil and chemical dispersant: Potential impacts of ocean acidification
Source: PLoS One. 2020 Jul 7;15(7):e0235473. doi: 10.1371/journal.pone.0235473 (PMC7340286; doi:10.1371/journal.pone.0235473)
Supplement: Supplementary file 3 [file pone.0235473.s001.docx]

**Supplementary Table 1:**							
**PAH composition and concentration in AGG and SSW fractions in both control and pCO2 conditions of all the treatments.**							
	**Treatment**	**Type**	**Naphthalenes**	**Biphenyl**	**Acenaphthylene**	**Acenaphthene**	**Fluorenes**
Control_A _SSW	*Control*	*SSW*	252.71	13.67	0.66	2.08	76
Control_B_SSW	*Control*	*SSW*	270.49	6.32	0	1.09	66.2
Control_C_SSW	*Control*	*SSW*	214.49	5.28	0.63	1.82	41.82
OA_A_SSW	*OA*	*SSW*	120.49	6.95	0.72	2.05	39.49
OA_B_SSW	*OA*	*SSW*	105.58	6.05	1.06	1.91	42.32
OA_C_SSW	*OA*	*SSW*	42.57	4.43	1.11	0.91	8.03
WAF_A_SSW	*WAF*	*SSW*	44645.15	615.89	23.83	29.05	1116.95
WAF_B_SSW	*WAF*	*SSW*	31815.26	447.35	14.6	21.41	718.68
WAF_C_SSW	*WAF*	*SSW*	31569.81	697.13	24.62	41.02	1312.13
OAWAF_A_SSW	*OAWAF*	*SSW*	34696.98	731.1	25.27	40.98	1285.07
OAWAF_B_SSW	*OAWAF*	*SSW*	32690.41	851.95	28.36	43.28	1358.87
OAWAF_C_SSW	*OAWAF*	*SSW*	26750.48	699.97	25.06	31.65	1285.13
DCEWAF_A_SSW	*DCEWAF*	*SSW*	1311.95	44.03	2.81	3.85	240.43
DCEWAF_B_SSW	*DCEWAF*	*SSW*	1348.09	38.95	2.78	3.03	240.07
DCEWAF_C_SSW	*DCEWAF*	*SSW*	1126.07	29.61	2.59	3.07	208.1
OADCWAF_A_SSW	*OADCWAF*	*SSW*	1295.28	38.42	2.7	2.75	183.44
OADCWAF_B_SSW	*OADCWAF*	*SSW*	1290.67	39.63	2.67	2.38	191
OADCWAF_C_SSW	*OADCWAF*	*SSW*	1346.41	39.38	2.28	3.94	218.64
Control_A_AGG	*Control*	*AGG*	3088.57	191.5	0	0	158
Control_B_AGG	*Control*	*AGG*	568.02	25.02	0	0	93.29
Control_C_AGG	*Control*	*AGG*	79.63	46.77	0	0	32.77
OA_A_AGG	*OA*	*AGG*	42.14	49.48	0	0	46.17
OA_B_AGG	*OA*	*AGG*	135.36	51.2	0	0	48.57
OA_C_AGG	*OA*	*AGG*	66.36	127.19	0	0	50.24
WAF_A_AGG	*WAF*	*AGG*	42590.8	495.19	117.2	63.74	15713.32
WAF_B_AGG	*WAF*	*AGG*	29475.54	729.52	84.43	50.73	9226.54
WAF_C_AGG	*WAF*	*AGG*	92761.5	2372.01	267.39	143.02	31243.86
OAWAF_A_AGG	*OAWAF*	*AGG*	52360.18	1957.5	167.31	101.02	22385.59
OAWAF_B_AGG	*OAWAF*	*AGG*	65906.84	3789.1	128.03	164.48	30511.39
OAWAF_C_AGG	*OAWAF*	*AGG*	47977.96	1922.69	130.17	81.71	11829.25
DCWAF_A_AGG	*DCEWAF*	*AGG*	8386.15	134.44	19.15	10.29	5684.69
DCWAF_B_AGG	*DCEWAF*	*AGG*	13403.28	248.6	123	46.86	8246.23
DCWAF_C_AGG	*DCEWAF*	*AGG*	11871.23	268.68	55.29	24.68	6262.61
OADCWAF_A_AGG	*OADCWAF*	*AGG*	10476.38	167.77	32.2	45.03	5987.28
OADCWAF_B_AGG	*OADCWAF*	*AGG*	10122.35	232.94	30.71	24.22	5402.58
OADCWAF_C_AGG	*OADCWAF*	*AGG*	10981.02	256.28	46.03	33.52	6041.2

Supplementary Table 1:							
PAH composition and concentration in AGG and SSW fractions in both control and pCO2 conditions of all the treatments.							
	Phenanthrenes/Anthracenes	Dibenzothiophenes	Fluoranthenes/Pyrenes	Benzo(a)anthracene	Chrysenes	Benzo(b)fluoranthene	Benzo(k)fluoranthene
Control_A _SSW	45.81	35.36	3.42	0	0	0	0
Control_B_SSW	64.35	31.86	2.18	0.14	1.19	0.03	0.09
Control_C_SSW	47.09	24.32	10.79	0.08	8.58	0.08	0.05
OA_A_SSW	26.15	17.89	4.97	0.1	0.05	0.06	0.06
OA_B_SSW	32.67	22.2	12.37	0.05	0.03	0.04	0.05
OA_C_SSW	25.93	83.59	13.1	1.25	3.39	3.59	3.5
WAF_A_SSW	2475.02	549.05	113.3	1.33	61.48	0	0
WAF_B_SSW	2771.06	598.62	114.66	1.57	49.91	0	0
WAF_C_SSW	2745.97	614.24	130.33	1.69	35	0.41	0.12
OAWAF_A_SSW	2851.85	605.6	131.33	1.72	64.93	0.36	0.25
OAWAF_B_SSW	2599.76	575.77	127	1.23	52.57	0	0
OAWAF_C_SSW	2710.96	616.59	123.89	1.31	53.5	0.18	0.34
DCEWAF_A_SSW	363.17	90.07	40.09	0.95	27.36	0.22	0.31
DCEWAF_B_SSW	423.45	86.1	68.39	1.18	30.63	0.42	0.42
DCEWAF_C_SSW	343.16	72.74	37.01	0.74	33.66	0.27	0.33
OADCWAF_A_SSW	347.58	121.17	36.55	1.31	32.84	0.71	0.35
OADCWAF_B_SSW	336.28	113.12	34.24	1.16	29.66	0.28	0.08
OADCWAF_C_SSW	432.93	151.63	101.4	2	93.82	1.03	0.18
Control_A_AGG	338.41	125.77	334.57	0	0	0	0
Control_B_AGG	3470.71	175.9	431.7	76.31	45.98	12.54	8.2
Control_C_AGG	146.21	46.39	151.13	0	0	0	0
OA_A_AGG	106.93	0	28.46	28.62	22.36	0	0
OA_B_AGG	187.77	31.93	492.87	0	0	0	0
OA_C_AGG	258.8	53.79	503.67	0	29.32	0	0
WAF_A_AGG	36878.55	9309.66	4237.41	95.33	5224.48	81.26	14.69
WAF_B_AGG	20491.98	3844.26	2404.47	153.04	3416.16	291.96	91.53
WAF_C_AGG	80108.28	15641.16	11069.02	508.02	14749.01	629.62	208.65
OAWAF_A_AGG	54753.02	9915.51	6461.68	276.07	7498.21	380.86	82.22
OAWAF_B_AGG	73988.82	13300.62	9452.95	224.16	9375.76	254.38	31.48
OAWAF_C_AGG	25589.09	4442.9	3064.03	160.14	3568.26	211.82	79.7
DCWAF_A_AGG	23888.81	5636.38	6449.22	218.93	11427.7	188.46	19.25
DCWAF_B_AGG	37708.88	7753.16	10845.81	253.36	16245.23	255.28	14.59
DCWAF_C_AGG	27009.93	5070.89	8202.82	261.06	13426.56	489.38	128.19
OADCWAF_A_AGG	28528.34	5724.56	8215.98	301.23	15111.69	310.67	68.39
OADCWAF_B_AGG	22189.66	4275.56	6700.78	182.19	10464.77	195.4	41.54
OADCWAF_C_AGG	25464.86	4773.29	7221.51	328.66	13765.55	405.79	264.41

Supplementary Table 1:						
PAH composition and concentration in AGG and SSW fractions in both control and pCO2 conditions of all the treatm						
	**Benzo(e)pyrene**	**Benzo(a)pyrene**	**Perylene**	**Indeno(1,2,3-c,d)pyrene**	**Dibenzo(a,h)anthracene**	**Benzo(g,h,i)perylene**
Control_A _SSW	0	0	0	0	0	0
Control_B_SSW	0	0.04	0	0	0	0
Control_C_SSW	3.73	0.05	0	0	0	0
OA_A_SSW	6.02	0.06	0	0	0	0
OA_B_SSW	0	0.08	0	0	0	0
OA_C_SSW	3.23	3.61	0	0	0	0
WAF_A_SSW	0	0	0	0	0	0
WAF_B_SSW	0	0	0	0	0	0
WAF_C_SSW	0	0	0.67	0	0	0
OAWAF_A_SSW	0	0	0.66	0	0	0
OAWAF_B_SSW	0	0	0	0	0	0
OAWAF_C_SSW	0	0	0	0	0	0
DCEWAF_A_SSW	0	3.92	0.91	0	0	0
DCEWAF_B_SSW	0	0	1.09	0	0	0
DCEWAF_C_SSW	0	0	0.76	0	0	0
OADCWAF_A_SSW	10.43	0.28	1.04	0.51	0.92	0.63
OADCWAF_B_SSW	23.41	0.12	0.83	0.42	0.39	0.4
OADCWAF_C_SSW	8.8	0.38	2.39	0.86	0.91	1.68
Control_A_AGG	233.67	25.1	0	0	0	0
Control_B_AGG	183.72	57.16	0	0	0	0
Control_C_AGG	0	0	0	0	0	0
OA_A_AGG	0	0	0	0	0	0
OA_B_AGG	0	0	0	0	0	0
OA_C_AGG	46.55	0	0	0	0	0
WAF_A_AGG	129.01	0	117.02	0	0	0
WAF_B_AGG	282.61	50.1	198.77	62.19	74.39	119.81
WAF_C_AGG	780.18	144.9	632.5	180.52	208.55	361.64
OAWAF_A_AGG	459.79	112.73	410.22	152.5	153.91	153.43
OAWAF_B_AGG	444.07	35.52	373.43	0	80.09	175.78
OAWAF_C_AGG	283.12	97.69	327.68	101.57	79.14	151.63
DCWAF_A_AGG	290.35	7.02	352.01	0	51.85	94.33
DCWAF_B_AGG	417.82	61.1	486.3	0	23.9	116.95
DCWAF_C_AGG	512.24	156.08	621.65	172.74	203.37	237.65
OADCWAF_A_AGG	433.65	75.05	436.54	86.43	129.3	145.28
OADCWAF_B_AGG	299.7	75.16	357.34	48.49	83.92	97.96
OADCWAF_C_AGG	541.81	182.24	628.23	110.52	141.26	243.83

Supplementary Table 1:

PAH composition and concentration in AGG and SSW fractions in both control and pCO2 conditions of all the treatments.

	**2-Methylnaphthalene**	**1-Methylnaphthalene**	**2,6-Dimethylnaphthalene**	**1,6,7-Trimethylnaphthalene**	**1-Methylphenanthrene**	
Control_A _SSW	49.57	24.14	34	9.55	4.95	
Control_B_SSW	14.26	9.01	15.91	7.51	5.96	
Control_C_SSW	18.08	10.16	9.95	1.93	3.01	
OA_A_SSW	20.16	12.84	11.81	4.19	1.79	
OA_B_SSW	14.59	13.57	10.82	4.34	3.33	
OA_C_SSW	9.2	6.41	5.96	2.94	1.68	
WAF_A_SSW	11279.99	10221.09	1184.73	239.14	259.9	
WAF_B_SSW	8082.28	7311.79	774.13	166.35	283.43	
WAF_C_SSW	7979.01	7301.42	1293.35	255.5	281.71	
OAWAF_A_SSW	8754.12	7877.3	1357.51	263.4	294.1	
OAWAF_B_SSW	8384.17	7624.15	1337.77	295.82	265.14	
OAWAF_C_SSW	6394.6	6076.87	1240.96	269.92	295.49	
DCEWAF_A_SSW	249.08	187.27	140.73	45.42	31.37	
DCEWAF_B_SSW	254.68	186.62	549.63	41.19	34.54	
DCEWAF_C_SSW	187.12	133.43	111.41	39.15	29.43	
OADCWAF_A_SSW	226.84	164.1	125.19	45.03	30.54	
OADCWAF_B_SSW	225.03	163.25	108.8	43.45	28.7	
OADCWAF_C_SSW	237.13	169.88	112.88	41.61	32.88	
Control_A_AGG	61.11	30.72	293.84	0	166.26	
Control_B_AGG	9.47	12.22	0	0	187.2	
Control_C_AGG	21.37	41.64	0	0	0	
OA_A_AGG	8.66	13.17	0	0	0	
OA_B_AGG	52.22	41.06	0	0	0	
OA_C_AGG	16.86	38.87	0	0	0	
WAF_A_AGG	712.39	2119.07	1989.31	2823.57	3733.35	
WAF_B_AGG	1003.85	2753.01	1728.67	1768.06	2199	
WAF_C_AGG	2077.95	6315.28	5534.81	6580.19	7316.37	
OAWAF_A_AGG	725.75	2548.79	2423.91	5272.89	5646.57	
OAWAF_B_AGG	850.98	2760.77	1905.06	6047.34	7319.04	
OAWAF_C_AGG	2633.71	7004.97	2866.24	2882.99	2629.79	
DCWAF_A_AGG	457	382.29	625.24	417.18	1021.94	
DCWAF_B_AGG	820.7	535.82	970.6	770.74	1559.5	
DCWAF_C_AGG	835.31	551.04	954.78	603.59	1171.54	
OADCWAF_A_AGG	528.68	511.6	801.87	546.43	1149.43	
OADCWAF_B_AGG	748.9	468.47	752.95	464	850.12	
OADCWAF_C_AGG	809.26	610.99	799.26	593.26	1131.15	
