# Supplementary material for: Diatom aggregation when exposed to crude oil and chemical dispersant: Potential impacts of ocean acidification
Source: PLoS One. 2020 Jul 7;15(7):e0235473. doi: 10.1371/journal.pone.0235473 (PMC7340286; doi:10.1371/journal.pone.0235473)
Supplement: Supplementary file 4 [file pone.0235473.s002.docx]

PAH	average	sd	ratio	ava	avb	cumsum	p
Phenanthrenes/ Anthracenes	0.107	0.030	3.494	35.599	189.561	0.176	0.[illegible]
Naphthalenes	0.069	0.041	1.666	109.423	167.606	0.29	0.[illegible]
Chrysenes	0.066	0.024	2.718	6.739	99.009	0.399	0.[illegible]
Fluorenes	0.057	0.021	2.749	24.382	108.764	0.493	0.[illegible]
Fluoranthenes/Pyrenes	0.052	0.016	3.134	9.107	82.067	0.579	0.[illegible]
Dibenzothiophenes	0.046	0.014	3.317	17.281	84.132	0.655	0.[illegible]
1-Methylphenanthrene	0.026	0.012	2.286	11.157	50.657	0.699	0.[illegible]
1-Methylnaphthalene	0.025	0.016	1.601	50.299	41.654	0.741	1
2-Methylnaphthalene	0.025	0.015	1.671	53.450	30.76	0.781	1
1,6,7-Trimethylnaphthalene	0.021	0.013	1.663	11.114	43.534	0.816	0.[illegible]
Perylene	0.013	0.004	3.336	0.665	19.863	0.839	0.[illegible]
2,6-Dimethylnaphthalene	0.013	0.009	1.412	23.746	39.84	0.861	0.[illegible]
Benzo(e)pyrene	0.013	0.003	3.978	0.919	19.747	0.882	0.[illegible]
Biphenyl	0.012	0.008	1.445	16.012	27.966	0.902	0.[illegible]
Benzo(b)fluoranthene	0.012	0.003	3.537	0.474	17.066	0.921	0.[illegible]
Benzo(a)anthracene	0.010	0.003	3.675	1.152	15.397	0.938	0.[illegible]
Benzo(g,h,i)perylene	0.008	0.003	2.62	0.227	11.786	0.951	0.[illegible]
Dibenzo(a,h)anthracene	0.006	0.003	2.243	0.211	9.34	0.962	0.[illegible]
[illegible]	[illegible]	[illegible]	[illegible]	[illegible]	[illegible]	[illegible]	[illegible]
